# The Interactomic Analysis Reveals Pathogenic Protein Networks in *Phomopsis longicolla* Underlying Seed Decay of Soybean

**DOI:** 10.3389/fgene.2018.00104

**Published:** 2018-04-03

**Authors:** Shuxian Li, Bryan Musungu, David Lightfoot, Pingsheng Ji

**Affiliations:** ^1^Crop Genetics Research Unit, United States Department of Agriculture, Agricultural Research Service, Stoneville, MS, United States; ^2^Department of Plant Biology, Southern Illinois University, Carbondale, IL, United States; ^3^Department of Plant, Soil, and Agricultural Systems, Southern Illinois University, Carbondale, IL, United States; ^4^Department of Plant Pathology, University of Georgia, Tifton, GA, United States

**Keywords:** *Phomopsis longicolla*, soybean, interactome, network, protein–protein interactions, pathogenicity

## Abstract

*Phomopsis longicolla* T. W. Hobbs (syn. *Diaporthe longicolla*) is the primary cause of Phomopsis seed decay (PSD) in soybean, *Glycine max* (L.) Merrill. This disease results in poor seed quality and is one of the most economically important seed diseases in soybean. The objectives of this study were to infer protein–protein interactions (PPI) and to identify conserved global networks and pathogenicity subnetworks in *P. longicolla* including orthologous pathways for cell signaling and pathogenesis. The interlog method used in the study identified 215,255 unique PPIs among 3,868 proteins. There were 1,414 pathogenicity related genes in *P. longicolla* identified using the pathogen host interaction (PHI) database. Additionally, 149 plant cell wall degrading enzymes (PCWDE) were detected. The network captured five different classes of carbohydrate degrading enzymes, including the auxiliary activities, carbohydrate esterases, glycoside hydrolases, glycosyl transferases, and carbohydrate binding molecules. From the PPI analysis, novel interacting partners were determined for each of the PCWDE classes. The most predominant class of PCWDE was a group of 60 glycoside hydrolases proteins. The glycoside hydrolase subnetwork was found to be interacting with 1,442 proteins within the network and was among the largest clusters. The orthologous proteins FUS3, HOG, CYP1, SGE1, and the g5566t.1 gene identified in this study could play an important role in pathogenicity. Therefore, the *P. longicolla* protein interactome (PiPhom) generated in this study can lead to a better understanding of PPIs in soybean pathogens. Furthermore, the PPI may aid in targeting of genes and proteins for further studies of the pathogenicity mechanisms.

## Introduction

Proteins in living organisms perform many functions by physically interacting with each other (Bork et al., 2004). Interactomes have been described as the genome-wide roadmaps of inferred protein–protein interactions (PPIs). Investigating the model of PPI can enhance our understanding of the cellular process and biological interactions within an organism. Interactomes of model organisms such as *Arabidopsis thaliana* (L.) and *Saccharomyces cerevisiae* (Meyen) were built using high-throughput experimental methodologies (Arabidopsis Interactome Mapping Consortium, 2011). Interactomes have also recently begun to expand to the non-model organisms by predictions based on orthology. Predicted interactomes in agronomically important organisms, such as *Citrus sinensis*, *Oryza sativa* (L.), *Glycine max* (L.) Merrill, and *Zea mays* (L.), have also provided valuable insight into disease resistance (Afzal et al., 2009; Musungu et al., 2015). In recent years, an abundance of PPI data has been developed through high-throughput technologies, including plant pathogens such as *Fusarium graminearum* (Zhao et al., 2009) that causes *Fusarium* head blight of both wheat and barley; *Gibberella* stalk rot of maize (Goswami and Kistler, 2004); and *Magnaporthe grisea* (He et al., 2008), cause of rice blast (Talbot, 2003; Dean et al., 2005).

*Phomopsis longicolla* T. W. Hobbs (syn. *Diaporthe longicolla*) is a fungal species of Ascomycota in the Diaporthaceae family. It is the primarily causal agent of Phomopsis seed decay (PSD) in soybean, *G. max* (L.) Merrill (Hobbs et al., 1985; Santos et al., 2011; Li et al., 2015a). This pathogen also causes stem lesion on velvet leaf plants (Li et al., 2001) and can live as an endophyte in the mangrove and Meliaceae plant species (Rhoden et al., 2012). *P. longicolla* has also been reported to produce a number of cytotoxic and antimicrobial secondary metabolites, such as dicerandrols and phomoxanthones (Isaka et al., 2001; Lin et al., 2010).

Soybean is one of the most important economic crops in the world with global production over 340 million metric tons (ASA, 2017). The soybean PSD disease results in poor seed quality and it is one of the most economically important seed diseases in soybean (Sinclair, 1993; Li, 2011). Management of PSD has been conducted using conventional tillage to reduce pathogen inoculum, rotation with non-host or non-legume crops, and early harvest (once soybeans have matured) to avoid late season wet weather. However, inconsistent reductions of PSD have been reported when those common agronomic practices were used. Fungicide treatments have also been an option to reduce PSD, but they were not always effective in controlling PSD (TeKrony et al., 1985; Wrather et al., 2004). Planting PSD-resistant cultivars is a cost-effective and long-term strategy to manage PSD. In past decades, research has been conducted to identify PSD-resistance sources by screening soybean germplasms, commercial cultivars, and breeding lines (Li et al., 2011, 2015c; Li and Chen, 2013; Li and Smith, 2016), investigating inheritance of resistance and genetic mapping of resistance to PSD (Zimmerman and Minor, 1993; Jackson et al., 2005, 2009; Smith et al., 2008), and breeding for resistant lines and cultivars (Minor et al., 1993; Pathan et al., 2009). However, information about mechanisms underlying the pathogenicity of *P. longicolla* on soybean is lacking. The prediction of PPI networks in *P. longicolla* has not been investigated and reported.

Although the genome of the *P. longicolla* isolate MSPL 10-6 has been sequenced (Li et al., 2015b, 2017), there are still many genes with unknown functions. Therefore, using the computational biology approach to analyze the interactome will help understand the different mechanisms underlying pathogenicity in PPI networks. Hence, the objectives of this study were to perform a genome-wide analysis of the predicted proteins interactome and to identify conserved global networks and pathogenicity subnetworks in *P. longicolla* causing PSD of soybean. This research will enhance our knowledge of the biology, pathogenicity, and protein interactions of *P. longicolla* and aid in developing improved disease management strategies for PSD.

## Materials and Methods

### Constructing a Protein–Protein Interaction Network for *Phomopsis longicolla*

The interolog method (Yu et al., 2004) was used to predict protein interactions in *P. longicolla*. The framework for the pipeline involved retrieving the protein sequences from NCBI and using the Inparanoid 4.1 software to infer one-to-one and many-to-many orthology. For one-to-one orthology selection, the proteins pairs with the most significant inparanoid orthology score were considered one-to-one. For the remaining proteins in the cluster, each of the protein pairs was considered to be in the many-to-many ortholog group. Afterwards, in house Python and R scripts were used to combine BioGrid data to allow for development of confidence values (CV).

The genome of a *P. longicolla* isolate MSPL 10-6 has been sequenced and the amino acid sequences of the *P. longicolla* proteins predicted to be encoded were retrieved from the National Center for Biotechnology Information (NCBI; Li et al., 2015b, 2017). The protein sequences of thirteen reference species, including eight eukaryotes (*Candida albicans, C. elegans*, *Drosophila melanogaster*, *Homo sapiens*, *Mus musculus*, *Rattus norvegicus*, *Saccharomyces cerevisiae*, *S. pombe*) and four prokaryotes (*Bacillus subtilis*, *Campylobacter jejuni*, *Escherichia coli*, and *Helicobacter pylori*) were retrieved from ENSEMBL www.ensembl.org/index.html (access date November 2011; Flicek et al., 2013) and NCBI http://www.ncbi.nlm.nih.gov (Geer et al., 2010).

Previous methodologies described by Geisler-Lee et al. (2007) and Musungu et al. (2015) were used in the Inparanoid 4.1 analysis. Briefly, the following organisms, *Bacillus subtilis*, *C. elegans*, *C. jejuni*, *D. melanogaster*, *E. coli*, *H. sapiens*, *M. Musculus*, *R. norvegicus*, *S. cerevisiae*, and *S. pombe*, were used as reference organisms in the Inparanoid pipeline (Koonin, 2005; Östlund et al., 2010). Inparanoid works by performing a proteome wide blast comparison between different organisms. It also allows for one-to-one and many-to-many predicted protein interactions to be inferred.

### Predicting *Phomopsis longicolla* Interactions From Conserved Orthologs

The interactome of *P. longicolla* was constructed from an all-inclusive analysis of physical interactions between proteins of *P. longicolla* that were predicted based on experimentally determined interactions for the organisms utilized in the study. For determining the protein interacting partners within the PPI network, the previously developed methods (Geisler-Lee et al., 2007; Musungu et al., 2015) were used to infer the unique interactions from a publically available interactome database (BioGRID, version 3.1.84^1^; Stark et al., 2006). The confidence value (CV), gene ontology, and the analysis methods described by Geisler-Lee et al. (2007) were used (**Supplementary Tables S1**, **S2**). Additionally, the gene ontology analysis used was the best BLAST hit in *F. graminearum* because the protein domain information for *P. longicolla* was not available (Güldener et al., 2006; Carbon et al., 2009). InterproScan analysis was also used for the genome to identify domains in the genome (Zdobnov and Apweiler, 2001). The presence of pathogenicity genes in the *P. longicolla* interactome was determined using the curated dataset from plant host interactions (Urban et al., 2016).

### Modeling *Phomopsis longicolla* Interactome Using Cytoscape

To visualize the PPI interactions from the network analysis, the *P. longicolla* protein data (**Supplementary File S1** and **Supplemental Table S1**) was used as the input file in the Cytoscape (version 3.5.1) analysis (Shannon et al., 2003; Cline et al., 2007).

### Cross Validation Analysis

**FIGURE 1:**
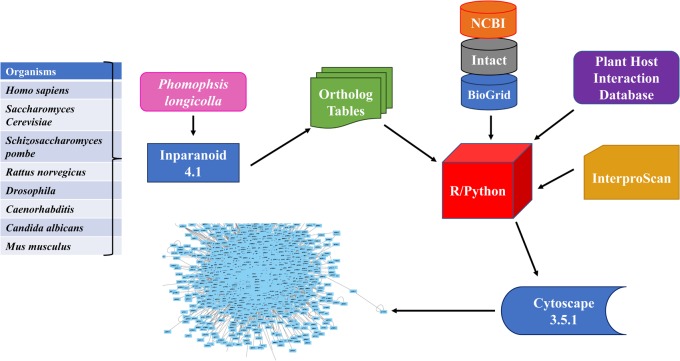
Flowchart for developing *Phomopsis longicolla* interactome. Generation of the interactome was accomplished by using publicly available resources (refer to section “Materials and Methods”) from multiple reference genomes. The ortholog prediction software Inparanoid was used for identification of orthologous proteins between *P. longicolla* and reference organism. The confidence values were calculated using R statistical language and Python. Additionally, verification was done with cross validation in R statistical language using domain information from EMBL Interproscan, Plant Host Interaction (PHI) data base and NCBI.

Cross validation was performed in R statistical language using the caret package doing *K*-fold cross validation (Kuhn, 2008). For feature selection we used the PFAM information available for each of the protein sequences after performing InterproScan. For the cross validation, positive interactions were identified using interlog method and the random interlog dataset was created from non-interacting proteins. Due to lack of experimentally determined data for *P. longicolla*, the PFAM domain information for the proteins was used. Rules for the features listed were:

(1) If the set of domains in Protein A {**domain A, domain B, domain C**} and Protein B {**domain A, domain B, domain C**} is true, then the value in matrix was set 1.
(2) If the set of domains in Protein A {**domain A, domain B, domain C, domain D**} and Protein B {**domain A, domain B, domain C**} are different, then the value in the matrix was set 2.
(3) If there were no domains or interaction found for the protein, then the interaction was set at 0.
(4) The methodology framework for this study was illustrated in **Figure 1**.

## Results

### General Features of the *Phomopsis longicolla* Interactome

**FIGURE 2:**
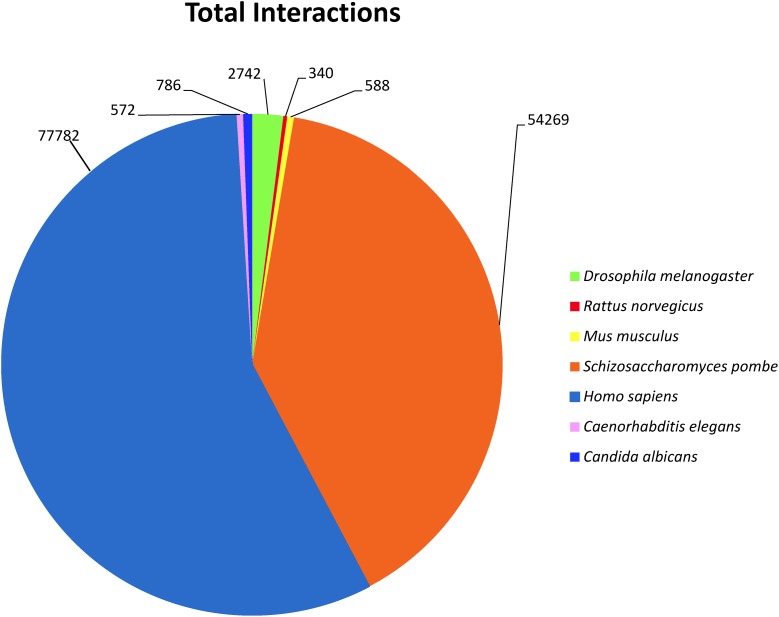
Analysis of reference organism proteins in PiPhom interactome. For each of the proteomes the distribution was calculated for their overall contribution for the total (One to One/Many to Many) interactome for the organisms *Caenorhabditis elegans*, *Candida albicans Drosophila melanogaster*, *Homo sapiens*, *Mus musculus*, *Rattus norvegicus*, *Saccharomyces cerevisiae*, and *Saccharomyces pombe* (*S. cerevisiae* was analyzed but not included in the figure due to large percentage of orthologous proteins).

To investigate predicted physical protein–protein interactions all the predicted proteins encoded by the *P. longicolla* genome were used. There were 215,255 unique PPIs among 3,868 of 16,595 predicted proteins. The relative contribution of each reference species to the predicted interactions is summarized in **Supplementary Table S1**. The resulting *P. longicolla* protein interactome (PiPhom) encompassed just 23% of the total proteome because the paralogous and duplicated genes from the genome were excluded. When duplicated genes were included in the prediction of the interactome, using a many-to-many ortholog matching method that allows the inclusion of paralogs, 50 *P. longicolla* proteins that were only in the many-to-many set, as well as 189 unique interologs, were added to the uniqueinteractome (**Table 1**). A premade Cytoscape formatted graphical visualization of *P. longicolla* interactome for this combined set of proteins was included (**Supplementary File S1**). In addition, contributions from each organism were highlighted (**Supplementary Table S1**), where *S. cerevisiae* had the largest contribution of total interactions including both “one to one” and “many to many” for the PPI data set (78%, **Figure 2**). *H. sapiens* contributed the second largest number (13%) of interactions to the PiPhom (**Supplementary Table S1**).

**Table 1:** Predicted protein–protein interactions in *Phomopsis longicolla*.

Orthology	Proteins	Interactions
Combined Total (One to One/Many to Many)	3863	624927
One to One Total	3818	307032
Many to Many Total	3821	317895
Combine Total Unique (One to One/Many to Many)	3863	244535
One to One Total	43	1737
Many to Many Total	50	189


### Validation and Network Analysis of Predicted *Phomopsis longicolla* Interactions

In order to determine the significance of the PiPhom interactome, analysis of conserved functional subnetwork models was conducted to determine if conservation of biological pathways in eukaryotes were present within the PiPhom interactome. The analysis of conservation indicated the subnetworks with the strongest confidences values which were similar to previously reported interactomes.

Confidence values (CVs) for each interaction in the PiPhom interactome are listed (**Supplementary Table S1**) and added to the network visualization (**Supplementary File S1**) as an edge feature. Interactions with a CV of 1 were ranked as a low confidence data set. They were identified in a single reference source using only one species and one experimental method. *P. longicolla* had 9,897 such interactions. The next level contained 191,407 interactions at the medium CV score of (2 ≤*x* ≤ 10). The high confident set contained 13,952 interactions with a CV (*x* > 10). The frequency profile of CV was similar to previous work where the large portion of unique interactions had medium confidence which is likely due to the high similarity between reference organisms. Due to novelty of the genome, *K*-fold 10 cross-validation was used with the domain information. From the *K*-fold, the best model for the data using the domain information inferred a 56% accuracy rating.

In the PiPhom interactome, many homo-interactions were identified. The top 20 conserved interactions in the PiPhom interactome were found to contain protein kinases and cellular machinery such as histone proteins (**Table 2**). The networks of *P. longicolla* displayed many of the core regulatory machineries in the cell, such as proteins involved in DNA-repair, zinc finger proteins, and heat shock proteins that were important in multiple eukaryotic systems and organisms (**Tables 2**, **3**). One of the expected pathways mined from the CV analysis of the network was the DNA repair machinery subnetworks (**Supplementary File S1**). The higher the CV, the greater the likelihood of the conserved interactions detected in PiPhom interactome.

**Table 2:** The highest confidence hetero-interactions in the predicted *Phomopsis longicolla* interactome^∗^.

Protein A	Protein B	Annotation A	Annotation B	CV
g14773.t1	g6302.t1	Protein kinase domain	Cyclin, C-terminal domain	10176
g399.t1	g482.t1	Protein kinase domain	CBS domain	7800
g11614.t1	g16409.t1	WD40 repeat	HORMA domain	5940
g1006.t1	g2595.t1	S-phase kinase-associated protein 1-like	Cullin, N-terminal	5472
g11614.t1	g5475.t1	WD40 repeat	Protein kinase domain	5115
g2595.t1	g878.t1	Cullin, N-terminal	Zinc finger, RING-type	5103
g13605.t1	g9923.t1	Small GTPase superfamily	CRIB domain	5080
g8719.t1	g8787.t1	Ubiquitin-conjugating enzyme E2	Zinc finger, RING-type	4896
g13046.t1	g8796.t1	MCM domain	MCM domain	4510
g770.t1	g7700.t1	Gtr1/RagA G protein	Gtr1/RagA G protein	4240
g13831.t1	g6437.t1	WD40 repeat	HEAT repeat	4074
g473.t1	g8682.t1	Septin	Septin	3696
g14053.t1	g15590.t1	Histone H3/CENP-A	Histone chaperone ASF1-like	3498
g16545.t1	g8796.t1	MCM domain	MCM domain	3476
g147.t1	g753.t1	RecF/RecN/SMC, N-terminal	RecF/RecN/SMC, N-terminal	3456
g651.t1	g9545.t1	Ubiquitin domain	von Willebrand factor, type A	3432
g3234.t1	g9687.t1	JAB1/MPN/MOV34 metalloenzyme	JAB1/MPN/MOV34 metalloenzyme	3366
g149.t1	g6302.t1	WD40 repeat	Cyclin, C-terminal domain	3320
g13046.t1	g16545.t1	MCM domain	MCM domain	3036
g10665.t1	g16545.t1	MCM domain	MCM domain	2916


**^∗^Unique one-to-one interactome interactions with the highest confidence values.**

**Table 3:** The most connected proteins in the predicted *Phomopsis longicolla* interactome.

Phomopsis Protein ID	Go-annotation	Interpro description	Count
g5566.t1	Uncharacterized protein	Uncharacterized protein	1391
g4900.t1	Uncharacterized protein	Actin family	1290
g12934.t1	Response to stress	Heat shock protein Hsp90 family	911
g14773.t1	ATP binding	Protein kinase domain	895
g9687.t1	Protein binding	JAB1/MPN/MOV34 metalloenzyme domain	821
g15300.t1	Ubiquitin-protein transferase activity	C2 domain	793
g671.t1	Intracellular	RNA recognition motif domain	791
g3518.t1	Intracellular protein transport	Importin-beta, N-terminal domain	783
g943.t1	Hydrolase activity	CDC48, N-terminal subdomain	705
g7778.t1	Vesicle-mediated transport	Synaptobrevin	702
g13329.t1	GTP binding	Septin	700
g7332.t1	Uncharacterized protein	Topoisomerase II-associated protein PAT1	685
g3598.t1	Iron ion binding	Cytochrome b5-like heme/steroid binding domain	683
g6853.t1	Zinc ion binding	Zinc finger, CCHC-type	679
g105.t1	ATP binding	Protein kinase domain	674
g76.t1	Calcium ion binding	EF-hand domain	653
g2704.t1	Metal ion binding	PPM-type phosphatase, divalent cation binding	638
g2424.t1	Ribosome binding	Translation elongation factor IF5A	637
g7340.t1	Uncharacterized protein	C2 domain	633
g10069.t1	Phosphoric ester hydrolase activity	SAC domain	628


**Number of predicted interacting protein partners for the unique interactome.**

Proteins in PiPhom interactome with a large number of interacting partners were found to be highly conserved (**Table 3**). The highly connected proteins were ubiquitous partners and co-factors such as cullins, scaffolding proteins, and proteins involved in degradation pathways. However, the protein with the highest connectivity was uncharacterized (g5566.t1), which had 1,391 different predicted protein partners. Additional analysis of the gene using InterproScan found it to be a cytochrome P450 domain containing gene. BLAST searching against *F. graminearum* failed, but mining the interactome of yeast inferred g5566.t1 encoded an ortholog of NAB2, a protein involved in RNA transport. Other highly interconnected conserved proteins, such as chaperonins, heat shock proteins, and members of large protein complexes were also identified (**Table 3**). Among both plants and animals those key protein complexes were conserved within the highly connected hubs. Interestingly, conserved interactions between histones, proteasome components, MutS type DNA repair proteins, and cytochromes were also found in PiPhom interactome. Thus, there is a similar hub pattern in *P. longicolla* to previously reported interactomes where there was a high degree of confidence. The hubs in the PiPhom interactome were similar to other eukaryotes even when comparing the small proteome to the large genomes with multiple divergent protein copies (Bork et al., 2004).

There was connection between phosphorylation of serine-threonine/tyrosine-protein kinases and transcription factors within the PiPhom interactome. Interestingly, the PiPhom interactome had orthologs to many of these signaling proteins. This may be due to the use of references proteomes from pathogens in the analysis, such as *C. albicans*, *S. cerevisiae* and *S. pombe*. An example could be seen with the (g15658.t1) FUS3, which is a signaling protein in the network with 224 proteins. Another protein was (g2599.t1) PMK1 which had a connectivity of 450 genes. Additional kinase modules included SNF1, STE11, STE12, HOG1, and RAC1 which were found to be hubs in abundant predicted interactions in PiPhom. Each of the connection contained larger than average degree of connectivity for these signaling proteins. When observing transcription factors, many of the conserved transcription factor complexes were present in the interactome, such as TFII complex and cell cycle proteins.

In addition, PiPhom contained pathways of interest to pathogenesis involved in pH, nitrogen metabolism, reactive oxygen species metabolism/catabolism and carbohydrate catabolism/metabolism which are conserved throughout the ascomycetes. These subnetworks represent modules of interest that have not previously been shown in other reported interactomes.

### Structural Analyses of the PiPhom Networks

**FIGURE 3:**
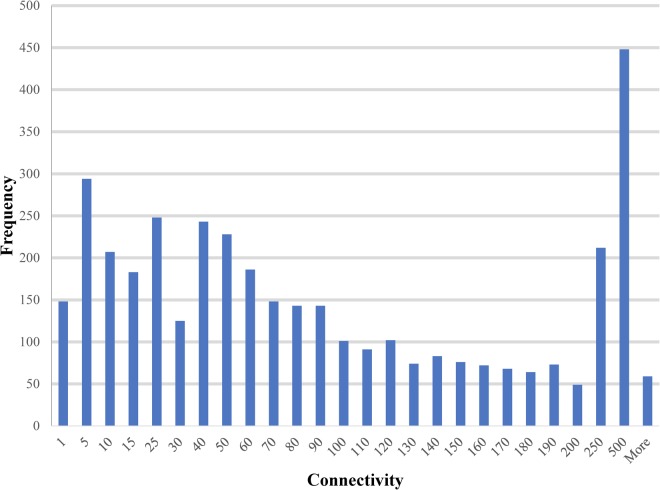
Degree of connectivity for unique proteins in the *Phomopsis longicolla* interactomes. The degree which is a measure of connectivity for vertexes and edges was analyzed for each of the proteins in the *P. longicolla* interactome.

The paths and trees in the PiPhom networks were measured by the structure analysis for mathematical properties, such as shortest paths, connectivity and circuits. The intermediate sized hubs were 10 to 100 interacting partners for the majority of proteins in PiPhom (**Figure 3**). The unique interactome had an average degree of connectivity of 110.496 neighbors per node.

The path length for PiPhom was defined by analyzing the average distance of Protein A to Protein B which was between 2 and 4 for the PPI network. Further analysis using the network analyzer module in Cytoscape indicated the mean path length to be 3.942 nodes (**Supplementary File S1**). Topology is a key indicator in inference of PPI networks. PiPhom shared similar network properties to previously reported interactomes and fungal specific structures within the network topologies.

### Fungal Gene Ontology Analysis of PiPhom

The interactome of *P. longicolla* was evaluated for enriched and depleted GO terms using the best *E*-value BLAST hits against *F. graminearum*. The PiPhom interactome was enriched significantly in GO: 0016301 (**Supplementary Table S2**). There was also a significant enrichment in ncRNA metabolic (GO: 0034660) processes within the network.

### Conserved Interactions Within the Network

**FIGURE 4:**
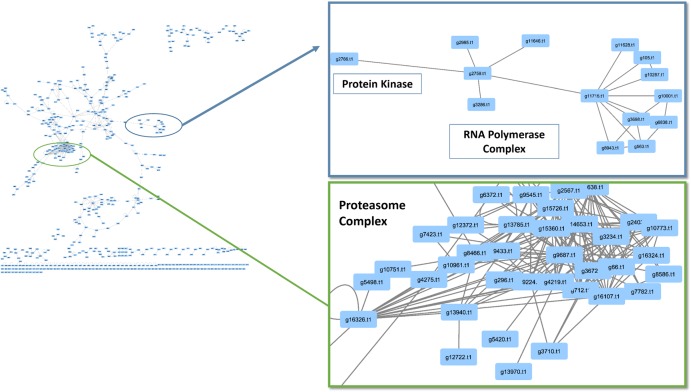
Conservation analysis of *Phomopsis longicolla* interactomes. Confidence value (CV) and at least 3 reference organisms were used to generate a subnetwork of protein and protein interactions of interest. Within the network there were proteasome complex, RNA-polymerase and small subsets of interactions.

When observing evolutionary conservation by species for the network, the largest subset of enriched interactions contained three or more reference organisms in the network. It has been demonstrated that conserved pathways are likely to be preserved throughout eukaryotes. There were 788 interactions identified in the high confidence set, in which the number or reference was greater than 3 and the CV value was greater than 10. The largest CV interaction seen in the network was (g14773.t1) protein kinase and (g6302.t1) cyclin, C-terminal domain which had a confidence value of 10,176 in the network (**Table 2**). The high confidence represented a portion of the network that had highly conserved interactions within the network (**Figure 4**). Within the stringent networks, proteins contained complexes such as the prefoldin, proteasome and vacuolar transport, which are all important mechanisms in eukaryotes.

### Plant Cell Wall Degrading Enzymes

**FIGURE 5:**
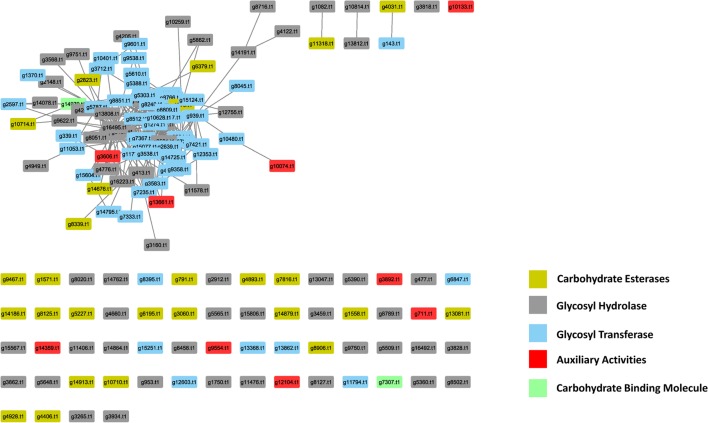
Analysis of the abundance of the cell wall degrading enzymes in the *Phomopsis longicolla* interactome. Five different classes were observed in the interactome: AA, auxiliary activities; CE, carbohydrate esterases; GH, glycoside hydrolases; GT, glycosyl transferase; CBM, carbohydrate binding molecule.

In the PiPhom interactome, there were 149 PCWDEs related proteins with 378 edges (**Figure 5**). The network captured five different classes of carbohydrate degrading enzymes, such as auxiliary activities (AA), carbohydrate esterases (CE), glycoside hydrolases (GH), glycosyl transferases (GT), and carbohydrate binding molecules (CBM). The most predominant class of PCWDEs was a group of 60 GH proteins which had been implicated in multiple pathogenicity studies (Daguerre et al., 2017). The smallest group of carbohydrate degrading proteins was the PCWDEs, belonging to the CBM family with just two proteins found in PiPhom. The two proteins were (g14970.t1), a CS domain containing 250 protein–protein interacting partners and (g7307.t1) a cysteine-rich secretory protein that had five interacting partners within the network.

### Pathogenicity Genes

**FIGURE 6:**
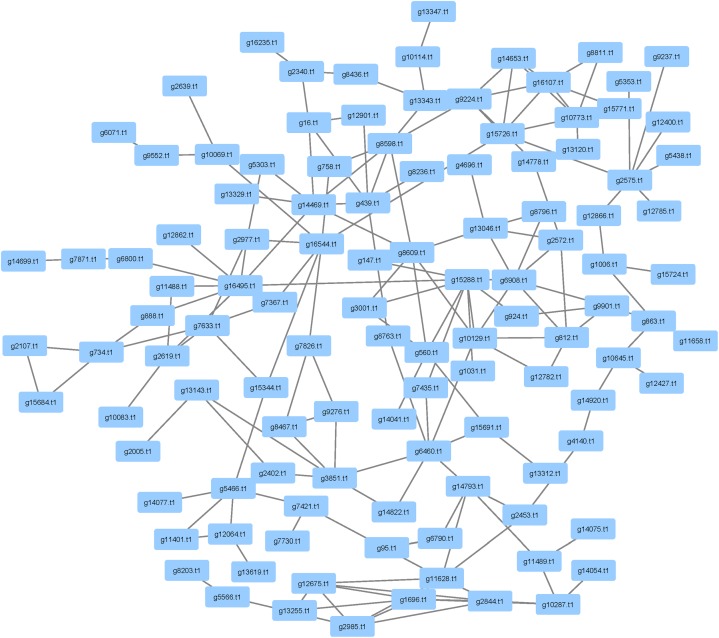
Analysis of pathogenicity genes using PHI-base and mined within the *Phomopsis longicolla* interactome. The network contains 477 proteins that were associated with pathogenicity. The network also contained several cell wall degrading enzymes including glycosyl transferase, auxiliary activities and carbohydrate esterases.

There were 1,414 pathogenicity genes that were identified in *P. longicolla* (**Supplementary Table S3**). Similarity among orthologous proteins was found for ascomycetes, basidiomycetes and eubacteria. Examples of taxa exhibiting hits within the curated data were *A. flavus*, *A. fumigatus*, *Alternaria alternata*, and *F. graminearum*. When focusing on proteins that have been inferred to be responsible for pathogenicity, 477 proteins were detected in the interactome (**Figure 6**). The high confidence data within pathogenicity network was 180 nodes and 257 edges.

## Discussion

Interologs were defined as a conserved interaction between a pair of proteins of a given organism which have interacting homologs in another organism (Yu et al., 2004). This method has been used to study and predict protein interactions successfully in other multiple organisms (Gu et al., 2011; Ho et al., 2012; Weßling et al., 2014), including but not limit to *O. sativa*, *C. elegans*, and *S. pombe*. Bioinformatic algorithms and programs, and corresponding parameters and weights used to produce *P. longicolla* interactomes in this study were similar to those used in *A. thaliana* (Geisler-Lee et al., 2007), *O. sativa* (Ho et al., 2012), and *Z. mays* (Musungu et al., 2015). In this study, a proteome-wide analysis of a predicted protein interactome using the interlog method was used to predict protein interactions in *P. longicolla* and developed to create resource for understanding the biology of the ascomycete *P. longicolla*. The PiPhom interactome can assist plant pathologists interested in possible gene-for-gene interactions and mycologists interested in possible industrial applications in agriculture. Additionally, the PiPhom may lead to a better understanding of this economically important soybean pathogen that causes seed decay. To date, analysis of the *P. longicolla* genome has been conducted to determine the genome features (Li et al., 2017) and comparative genome study with other soybean ascomycete pathogens (Li and Musungu, unpublished). The primary goal of the study was to predict PPIs and gain a functional understanding of proteins involved in the developmental processes, plant cell wall degrading enzymes (PCWDEs) and pathogenicity proteins which are important components of *P. longicolla.* In our previous study, PCWDEs encoded within the *P. longicolla* genome were determined (Li et al., 2017). In this study, a cysteine-rich secretory protein (g7307.t1) that had five interacting partners within the network was identified. The cysteine rich protein was an interesting conserved discovery in the network because many of these proteins have been shown to function as secretory proteins in fungi and oomycetes, such as *Phytophthora cactorum*, *Leptosphaeria maculans*, and *F. oxysporum* (Sperschneider et al., 2015). Using the Systems Biology approaches, networks among families of PCWDEs were identified (Li et al., 2017). Additionally, proteins that represented a graphical significance within the network through degree of connectivity were characterized.

The level of CV can be used as a filter to identify true hypotheses and reduce the false positives when the data is used to build networks. Many previously reported common hetero-interactions were often the most abundant interactions in PPIs (Musungu et al., 2015). However, that was not the case in the PiPhom interactome, in which many homo-interactions were identified. The networks of *P. longicolla* displayed many of the core regulatory machineries in the cell that were important in multiple eukaryotic systems and organisms. One of the expected pathways mined from the CV analysis of the network was the DNA repair machinery subnetworks, which were conserved throughout eukaryotes (Liu et al., 1999; Ohbayashi et al., 1999) and recovered from similar interactomes, such as *S. cerevisiae* (Yu et al., 2008), *Z. mays* (Musungu et al., 2015), and *A. thaliana* (Geisler-Lee et al., 2007) among others. The higher the CV, the greater the likelihood of the conserved interactions detected in PiPhom interactome.

Connectivity in computational biology has been demonstrated to aid in the generation of hypotheses for targeted pathogenicity analysis. This has been demonstrated in multiple interactome studies when working on systems with minimal biological information. In the PiPhom interactome, the highly connected proteins were ubiquitous partners and co-factors such as cullins, scaffolding proteins, and proteins involved in degradation pathways. This overlapped with the previous interactome studies, such as *A. thaliana*, *Z. mays*, and *P. patens*, which had highly conserved pathways represented by cullins. The highly connected interactions are likely to have a large degree of connectivity since it is evolutionarily conserved (Evlampiev and Isambert, 2008).

The enrichment of the PiPhom protein network led to identification of pathogenesis pathways, such as nitrogen metabolism, carbohydrate degrading enzymes and cell to cell signaling processes. It has already been demonstrated that mitogen activated protein kinases are important in the pathogenicity of multiple pathogens such as *F. graminearum*, *F. solani*, and *M. grisea* (Xu and Hamer, 1996; Di Pietro et al., 2001; Jenczmionka et al., 2003; Ramamoorthy et al., 2007). Moreover, a total of 477 pathogenicity-associated proteins were detected in the PiPhom interactome. The abundance of pathogenicity factors was likely due to yeast data as well as the addition of the pathogen *C. albicans* in which 80 of 478 proteins were inferred within pathogenicity networks. This is an abundant amount in comparison to previous studies where about 100 pathogenicity proteins were detected for the *M. grisea* PPI (He et al., 2008). Within the network, there were common pathogenicity genes, which are seen in other ascomycetes. For example, STE11 was associated with pathogenicity in *Botrytis cinerea* and *F. graminearum* (Izumitsu et al., 2009; Leroch et al., 2013; Gu et al., 2015). Connection between phosphorylation of serine-threonine/tyrosine-protein kinases and transcription factors was present within the interactome. Those pathogenic proteins have been used as targets for targeted mutagenesis (Caracuel et al., 2003; Shimizu et al., 2003; Ramamoorthy et al., 2007).

Analysis of orthology is a key concept during interactome analysis of protein–protein interactions. This is because interactions are likely to occur if there is conservation through different organisms which can be attributed to fitness of organism or random mutations (Lynch, 2007). These features of the interlog method have been demonstrated in previous interactomes such as *A. thaliana*, *Z. mays, H. sapiens*, and *S. cerevisiae*. Additionally, because our dataset is made up of interactions from *S. cerevisiae*, multiple developmental pathways in important life cycles of plant pathogens were identified. This was highlighted in the results with proteins such as FUS3 which has been implicated in pathogenesis in fungi such as *A. alternata*, *F. oxysporum*, and *M. oryzae* (Wilson and Talbot, 2009; Lin et al., 2010; Pareek and Rajam, 2017). Moreover, the HOG1 protein was identified, which was initially characterized and conserved in *S. cerevisiae* to be involved in osmotic signaling. It has also been demonstrated in multiple fungi to be involved in the regulation of pathogenesis. For example, in *Zymoseptoria tritici*, a hemibiotrophic pathogen, when HOG1 was targeted for knockout it displaced a loss in pathogenicity (Mehrabi et al., 2006). This was additionally seen in pathogens such as *Penicillium digitatum*, *Magnaporthe oryzae* and several others (Motoyama et al., 2008; Wang et al., 2014).

The PiPhom interactome was built using preexisting data from the biogrid and resembles other inferred networks built by the same methodology. The connectivity of PiPhom differed from previous interactomes like *Zea mays*, *Physcomitrella patens*, *D. melanogaster*, and *O. sativa* (Giot et al., 2003; Stark et al., 2006; Gu et al., 2011; Musungu et al., 2015; Schuette et al., 2015). The characteristic path length of PiPhom differed from the previously reported interactomes for organisms, such as *A. thaliana* (3.4), *S. cerevisiae* (2.6), *Stegodyphus mimosarum* (2.5) and *H. sapiens* (between 1 and 3; Gursoy et al., 2008; Taylor et al., 2009; Chen et al., 2012; Schuette et al., 2015; Wang and Jin, 2017). When contrasting the PiPhom with previous plant, animal, and fungal work, there was an abundance of signaling proteins in the conserved interactions, suggesting a difference in wiring vs. other fungal species. In contrast, the PiPhom conserved subnetwork modules were comprised of many of the pathways previously found in other interactomes, such as ubiquitination, methylation, pheromone signaling, developmental pathways and chromatin remodeling (Mosca et al., 2012). Thus the similarity between PiPhom and previous interactomes produced by similar methodology leads to increase in confidence in the novelties discovered (Geisler-Lee et al., 2007; Gu et al., 2011; Musungu et al., 2015). Furthermore, the other proteins that were found in the network will become targets for in-depth lab studies. Scientists who are interested in a particular PCWDE family would have the ability to mine the network. For example, the g5566t.1 gene that was identified to represent the highest degree of connectivity was initially identified as uncharacterized and containing cytochrome P450 domain information. While multiple genes have been identified to be involved with cytochrome P450 and pathogenicity for other pathogens, the protein sequence would have been initially missed because it was returned as uncharacterized during the initial BLAST analysis against *F. graminearum*. Utilizing the metadata in PiPhom, the yeast one-to-one ortholog NAB2 was inferred to be the closest ortholog of the g5566t.1 protein, partly because it had the largest degree of connectivity within the network. The protein was essential for cell viability in yeast (Anderson et al., 1993). Its primary function was inferred to be involved in RNA transport and confirmed primarily in yeast. However, mutations in *A. oryzae* have been able to show that knocking out of the gene altered the ability of the pathogen to grow compared to wild type (Yamada et al., 1999).

In addition, the interactions are informative for study of other biological processes due to the similarity between PhiBase proteins and other pathogens. From the analysis against the pathogen host interaction (PHI) database genes, multiple BLAST hits on virulence and pathogenicity factors were shared among ascomycetes. PiPhom will be used to select for genes of interest. For example, some of the orthologous proteins identified included CYP1, SGE1, PMK1 and many others from fungi and bacteria. The CYP1 and SGE1 have been characterized and previously shown to be involved in pathogenicity (Winnenburg et al., 2006). Another feature of the PiPhom interactome can be used to filter/identify the non-virulence and non-pathogenic hits that are stored within PHI database.

Overall, PiPhom showed several novel interactions specific to *P. longicolla*. This is likely due to the close ortholog overlap for *P. longicolla* proteins vs. yeast proteins within the network. The PiPhom interactome appeared different from previous studies of plant interactomes, such as maize (Musungu et al., 2015), in which the amount of interactions generated was limited by plant specific data.

## Conclusion

The PiPhom interactome generated from this study provides a valuable resource for understanding the complexity of pathogenicity in *P. longicolla*. The orthologous proteins, such as FUS3, HOG, CYP1, SGE1, PCWDE, and the g5566t.1 gene identified in this study could play an important role in pathogenicity of *P. longicolla*. This research enhances our knowledge of the biology, pathogenicity, and protein interactions of *P. longicolla* and aids in developing improved strategies for managing PSD. Moreover, the PiPhom interactome can also lead to a better understanding of PPIs in soybean pathogens. Furthermore, the PPI may aid in targeting of genes and proteins for further studies of pathogenicity mechanisms.

## Author Contributions

SL conceived and led the project, interpreted results and wrote the manuscript. BM analyzed data and wrote the manuscript. DL and PJ provided suggestions for the project and edited the manuscript. All authors reviewed and approved the final version of the manuscript.

## Conflict of Interest Statement

The authors declare that the research was conducted in the absence of any commercial or financial relationships that could be construed as a potential conflict of interest.

## Footnotes

1 www.thebiogrid.org

## Supplementary Material

FILE S1 Cytoscape PiPhom. This file contains the PiPhom predicted interactome as a Cytoscape ver 3.5.1 format file. Interactions shown are indicated and filterable by orthology (many-to-many and one-to-one), confidence value (numerical), and connectivity. Each of subnetworks contains cell wall degrading enzymes, parthenogenesis genes and conserved proteins.

TABLE S1 PiPhom Interactome Data Tables. The Master Interactome sheet contains all interolog predictions for the *Phomopsis longicolla* interactome using either many to many or one to one orthology. The supplemental data also contains the connectivity numbers for each of the interlog pairs for the interactome calculated from the master interactome. The dataset also includes the raw tables that were used within the publication. A Node Property sheet provides annotation and shows the number of interactions each proteins has in either the unique or combined datasets. The supplemental dataset also contains the unique data sheet for uploading data into Cytoscape for producing Supplemental File S1.

TABLE S2 gProfiler Results. Gene Ontology analysis was run on the proteins in the *Phomopsis longicolla* interactome using *Fusarium graminearum* orthologs. The significant pathways that were enriched had *p*-value < 0.05 to be considered an enriched pathway.

TABLE S3 *Phomopsis longicolla* Blast Results against Phibase. This supplemental table contains the results of Phibase database blast against the *P. longicolla* interactome. It shows *P. longicolla* proteins from the interactome that are inferred to be involved with virulence, non-virulence and pathogenicity factors.
